# Secular Trends in Time-of-Day of Energy Intake in a Chinese Cohort

**DOI:** 10.3390/nu14102019

**Published:** 2022-05-11

**Authors:** Xiaoyun Song, Huijun Wang, Chang Su, Zhihong Wang, Jiguo Zhang, Gangqiang Ding, Bing Zhang

**Affiliations:** 1 National Institute for Nutrition and Health, Chinese Center for Disease Control and Prevention, Beijing 100050, China; sxydljk@126.com (X.S.); wanghj@ninh.chinacdc.cn (H.W.); suchang@ninh.chinacdc.cn (C.S.); wangzh@ninh.chinacdc.cn (Z.W.); zhangjg@ninh.chinacdc.cn (J.Z.); dinggq@chinacdc.cn (G.D.); 2 National Health Commission Key Laboratory of Trace Element Nutrition, Beijing 100050, China

**Keywords:** time-of-day, energy intake, snacking, trend, cohort

## Abstract

Few studies have described the status and change of time-of-day of energy intake on a population level. This study aims to investigate the secular trend in time-of-day of energy intake using a Chinese cohort, and to examine demographic disparities in trends. A total of 20,976 adults with at least two waves of dietary data in the China Health and Nutrition Survey (CHNS 1991–2018) were included. A multilevel linear mixed model was applied to the energy proportions of breakfast, lunch and dinner. A multilevel Tobit regression model was applied to the energy proportion of morning snack, afternoon snack and evening snack. Time-demographic interaction terms were tested to examine demographic disparities in the trends. From 1991 to 2018, the marginal mean of the energy proportion of breakfast experienced first a falling and then a rising trend, and the marginal mean of energy proportions of lunch and dinner both presented first a rising and then a falling trend. The marginal means of all snacks took on a rising trend. Significant time-demographic interactions were observed for energy proportion of each eating occasion. On average, female, older and rural people tended to have a higher energy proportion at breakfast and lower energy proportion at lunch and dinner. Female, younger and urban people tended to have higher snack energy proportions. The time-of-day of energy intake has first shifted towards later in the day and then towards a balanced meal pattern in this Chinese cohort. Demographic disparities were observed in both the secular trend and the mean level of energy proportions of eating occasions. The health implications of such meal patterns warrant further investigation.

## 1. Introduction

While cardiometabolic diseases are rising worldwide, meal pattern has been suggested as a factor that could lead to weight gain and adverse cardiometabolic outcomes [1]. Previous studies have suggested that eating a larger proportion of energy in the later part of the day not only influences total daily energy intake [2], but also has close relationship with higher risk of obesity, type 2 diabetes and metabolic diseases [3,4,5,6].

Some studies have described the status and change of time-of-day of energy intake on a population level with varied results. One cross-sectional study [7] characterized meal patterns across ten European countries using dietary data collected during 1995–2000 in the European Prospective Investigation into Cancer and Nutrition calibration study. Pronounced differences in lunch and snack energy proportions were found between different countries. Lunch energy proportion was 41–45% for men and 38–43% for women in Mediterranean countries compared with 20–26% for men and 16–27% for women in central and northern countries. Likewise, snack energy proportions were 10–17% for men and 13–20% for women in Mediterranean countries compared with 23–35% for men and 24–34% for women in central and northern countries. Little difference was found for dinner energy proportion across countries. An American cross-sectional study [8] showed a trend of eating late in free-living adults, with less than 25% of daily energy consumed before noon and over 35% after 6 p.m. Data from the National Health and Nutrition Examination Survey (NHANES) [9] found that both breakfast and dinner energy proportions declined from NHANES 1971–1975 to NHANES 1999–2002 for American adults, while snack energy proportion increased from 19.7% in 1971–1975 to 20.8% in 1999–2002. Another study using a British birth cohort [10] described a decline in the proportion of energy and macronutrients consumed at lunch between 1982 and 1999, which was compensated for by an increase of energy in the mid-afternoon and evening. Previous studies have also reported differences in eating behaviors between genders, age groups and socio-economical levels [11,12,13,14], suggesting possible demographic disparities in time-of-day of energy intake.

Although studies conducted in the western countries suggested changes of meal patterns over time, there is still a gap concerning time trend in meal patterns in the Asian population. With the rapid economic growth and social change over the past two decades, the eating behaviors of the Chinese population have undergone changes [15]. Nationwide surveys conducted in China documented that the prevalence of breakfast skipping, lunch skipping, and dinner skipping were 3.2%, 1.7% and 0.6% in 2002 [16], and 3.1%, 1.1%, and 0.2% in 2010–2012 [17]. One previous study showed that snacking prevalence across all age groups of the Chinese population increased from 8.7–23.8% to 35.6–58.8% from 1991 to 2009 [18]. However, to the best of our knowledge, no study has explored the trend in time-of-day of energy intake for the Chinese population, and whether these trends differ by demographic characteristics is unknown.

Therefore, using longitudinal data from ten waves of the China Health and Nutrition Survey (CHNS 1991–2018), the present study aimed to (1) investigate the secular trends in energy proportions of breakfast, lunch, dinner and snacks in a Chinese adult population; and (2) to determine whether these trends differed by gender, age group and geographical region.

## 2. Materials and Methods

### 2.1. Study Participants

The China Health and Nutrition Survey (CHNS) is a population-based longitudinal survey in China, ongoing since 1989. Follow-up surveys carried out in 1991, 1993, 1997, 2000, 2004, 2006, 2009, 2011, 2015, 2018 collected dietary, anthropometric, clinical, and all other individual as well as household and community data. More details regarding the CHNS are provided in the previous article [19].

**Figure 1:**
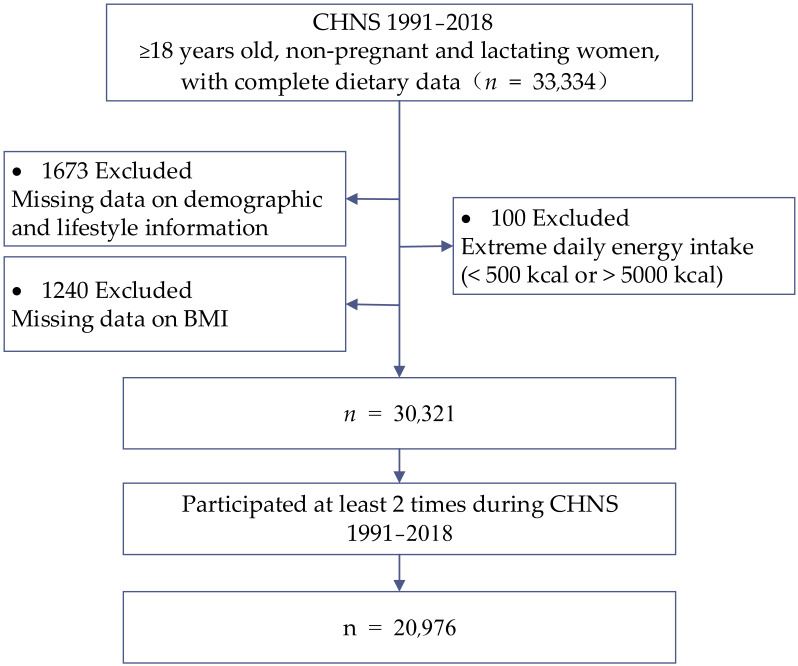
Flow chart of study population.

Our study included 10 waves of survey data (CHNS 1991, 1993, 1997, 2000, 2004, 2006, 2009, 2011, 2015, and 2018). Participants aged over 18 years-old who participated in at least 2 rounds during wave 1991 to 2018 with complete dietary data and anthropometric information were included in our analysis (*n* = 20,976). Figure 1 presents the flow chart of participant selection. The main reason for the drop-out of participants was moving out of the original community with the process of urbanization.

### 2.2. Dietary Assessment

Details of dietary assessment in CHNS have been described previously [20]. In the present study, dietary data were collected by three consecutive 24 h recalls (two weekdays and one weekend). Briefly, in each wave of CHNS, participants were interviewed by well-trained field interviewers to recall information on types and amounts of food consumed at each eating occasion during the previous 24 h. There were six pre-defined eating occasion response options in the CHNS 24 h recall: breakfast, lunch, dinner, morning snack, afternoon snack, and evening snack, from which participants self-reported the type of each eating occasion during the day. In the present study, amounts of every condiment (such as oil, salt, soy sauce) were collected through the weighing and measuring technique during the same three-day period. Energy intake from both food and condiment at each eating occasion was calculated by the China Food Composition.

### 2.3. Energy Proportion of Each Eating Occasion

In the present study, the energy proportions of 9 eating occasions were calculated: breakfast, lunch, dinner, morning snack, afternoon snack, evening snack, morning period (breakfast + morning snack), afternoon period (lunch + afternoon snack), and evening period (dinner + evening snack). Firstly, energy proportion at each eating occasion was calculated for each recall day through dividing energy intake at each eating occasion by daily total energy intake. Then, the energy proportion of each eating occasion was averaged across their consumption days during the survey period to obtain mean estimates. For simplicity, breakfast EI%, lunch EI%, dinner EI%, morning snack EI%, afternoon snack EI%, evening snack EI%, morning EI%, afternoon EI%, and evening EI% were used to represent energy proportions of breakfast, lunch, dinner, morning snack, afternoon snack, evening snack, morning period, afternoon period, and evening period, respectively in this study.

### 2.4. Covariates

We assessed covariates between 1991 and 2018. The following measures were considered covariates: Age group (18–59 years, ≥60 years); gender (male, female); geographic region (urban and rural); annual per capita household income (yuan/year); educational level (low [i.e., completed primary school], medium [i.e., completed middle school], high [i.e., completed high school and above]; total physical activity, calculated into a metabolic equivalent of task (METs h/week) based on the Compendium of Physical Activities [21]; smoking (non-smoker and current smoker); alcohol drinking (non-drinker and current drinker); marriage status(at marriage, other status); chronic disease history (yes [i.e., ever diagnosed with hypertension or cardiovascular disease or diabetes or cancer], no); community urbanicity index, calculated based on 12 multidimensional components including physical, social, cultural and economic environment of the community [19]; total energy intake; body mass index (BMI, kg/cm^2^). Demographic and lifestyle information were interviewed by well-trained field interviewers in each survey. Height was measured to the nearest 0.1 cm using height tape (model 206, SECA). Body weight was measured to the nearest 0.1 kg using a body fat meter (BC601, Tanita). Body mass index (BMI) was calculated as weight (kg)/height (m)^2^. Covariates in each wave were adjusted in the present study.

### 2.5. Statistical Analysis

For the trend analysis of breakfast EI%, lunch EI% and dinner EI%, a multilevel linear mixed model was applied. For trend analysis of morning snack EI%, afternoon snack EI% and evening snack EI%, a multilevel Tobit regression model was applied, because the value of morning snack EI%, afternoon snack EI% and evening snack EI% showed a high percentage of zero due to a relatively large share of participants not reporting any snack on the day of survey. Tobit regression model could handle dependent variables with an excess of zeros and an absence of negative values by censoring dependent variables to zero [22]. The positive value of morning snack EI%, afternoon snack EI% and evening snack EI% were log-transformed due to non-normality before applying the multilevel Tobit regression model.

To test the trend of time-of-day of EI% over 10 waves of measurements, time was treated as a continuous variable (continuously in years) in all models. Based on preliminary descriptive statistics, both linear and quadratic time terms were examined for each outcome. Age, gender, educational level, geographical region, total physical activity, smoking, alcohol drinking, marriage status, annual per capita household income, community urbanicity index, chronic disease history, total energy intake, and BMI were included as covariates. The fully adjusted model was used to estimate marginal mean of breakfast EI%, lunch EI%, dinner EI%, morning snack EI%, afternoon snack EI% and evening snack EI%, respectively.

To test the demographic disparity in the secular trend, possible time-demographic interaction terms were tested in a multilevel linear mixed model and multilevel Tobit regression model. Furthermore, the time trend in each demographic subgroup was also tested and marginal means were estimated by the fully adjusted model.

All the analyses were conducted in Stata 15SE (StataCorp., College Station, TX, USA). *p* < 0.05 was considered statistically significant.

## 3. Results

### 3.1. Characteristics of Study Sample in the CHNS 1991–2018

As shown in Table 1, the percentages of participants aged ≥60 years, female, married, living in urban areas, with medium to high educational level, and diagnosed with chronic diseases increased over time. The percentages of current smokers and current alcohol drinkers declined over time. Both total physical activity and energy intake decreased from 1991 to 2018, while BMI increased from 1991 to 2018.

### 3.2. Secular Trends of Energy Proportion of Eating Occasions

The secular trends and marginal mean of energy proportion of eating occasions were presented in Table 2. Breakfast EI% decreased by 1.4% between 1991 and 2000, and then increased by 4.0% by 2018. Lunch EI% increased by 0.2% between 1991 and 1997, and then decreased by 2.7% by 2018. Dinner EI% increased by 0.2% between 1991 and 1997, and then decreased by 2.5% by 2018. Log-transformed morning snack EI%, afternoon EI% and evening EI% increased from 0.01%, 0.02%, 0.03% to 0.37%, 0.43%, 0.42% from 1991 to 2018, respectively. EI% of the morning period (breakfast EI% + morning snack EI%) decreased between 1991 and 2000, followed by an increase between 2004 and 2018. On the contrary, both EI% of the afternoon period (lunch EI% + afternoon snack EI%) and the evening period (dinner EI% + afternoon snack EI%) increased between 1991 and 2000 and decreased by 2018.

### 3.3. Interactions between Time Variable and Demographic Variables

Table 3 showed the coefficients and *p* value of the interaction terms. On average, the rate of change in breakfast EI% was faster for female (β = 0.07, *p* = 0.019), ≥60 y (β = 0.09, *p* = 0.014) and urban participants (β = −0.24, *p* < 0.001). The rate of change in lunch EI% was on average faster for rural participants (β = 0.23, *p* < 0.001). The rate of change in dinner EI% was on average faster for 18–59 y (β = −0.09, *p* = 0.009) and rural participants (β = 0.003, *p* = 0.003). The morning snack EI% changed faster for female (β = 0.05, *p* = 0.024) and urban participants (β = −0.07, *p* = 0.003). The afternoon snack EI% changed faster for ≥60 years old (β = 0.12, *p* < 0.001) and rural participants (β = −0.001, *p* = 0.021), and the evening snack EI% changed faster for ≥60 years old (β = 0.07, *p* < 0.001).

### 3.4. Secular Trends of Energy Proportion of Eating Occasions by Demographic Subgroups

**Figure 2:**
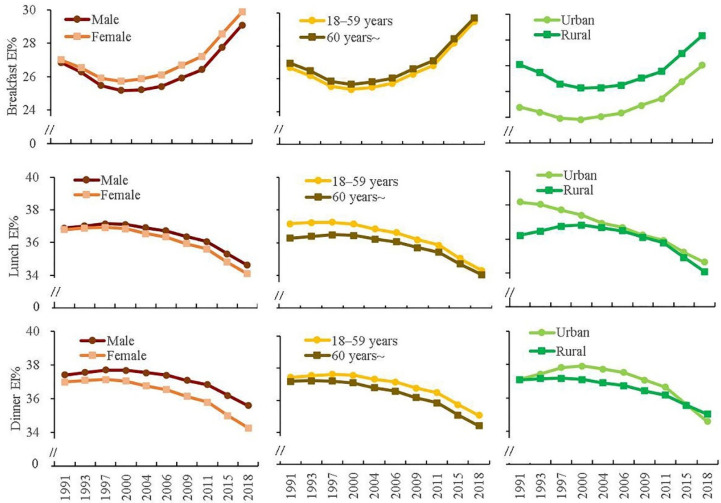
Marginal means of energy proportion of breakfast, lunch, dinner by gender, age-group and location ^1^. ^1^ Marginal mean was calculated from multilevel linear mixed model. Models were adjusted for age, gender, educational level, geographical region, total physical activity, smoking, alcohol drinking, annual per capita household income, community urbanicity index, chronic disease history, total energy intake, and BMI.

**Figure 3:**
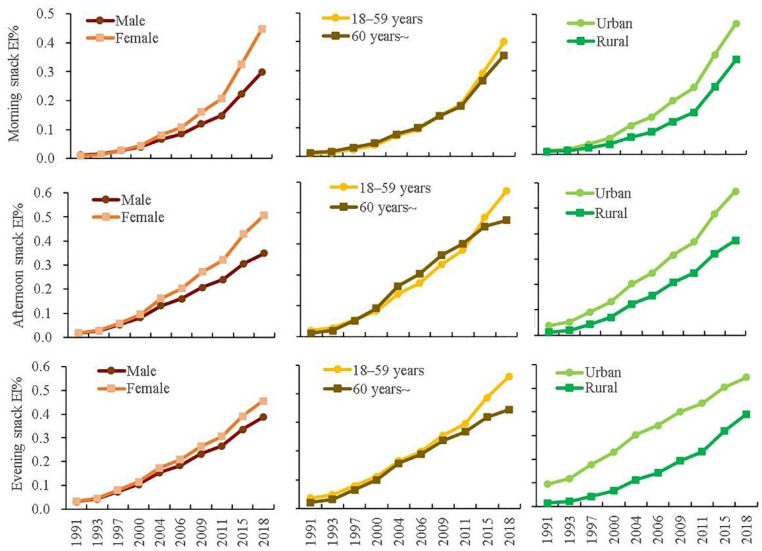
Marginal means of energy proportion of morning snack, afternoon snack, evening snack by gender, age-group and location ^1^. ^1^ Log-transformation was done to improve normality. Marginal mean was calculated from multilevel Tobit regression model. Models were adjusted for age, gender, educational level, geographical region, total physical activity, smoking, alcohol drinking, annual per capita household income, community urbanicity index, chronic disease history, total energy intake, and BMI.

The linear and quadratic trends of the EI% of eating occasions by different genders, age groups and locations are shown in Table 4. The marginal means of eating occasions of each subgroup were presented in Tables S1 and S2. Figure 2 and Figure 3 illustrated these marginal means.

With respect to the secular trends of the EI% of eating occasions in different subgroups, breakfast EI% in all subgroups deceased from 1991 to 2000, followed by an increase by 2018. Both lunch EI% and dinner EI% in all subgroups, expect for lunch EI% in the urban group, increased from 1991 to 2000, then decreased by 2018. Lunch EI% in the urban group showed a decreasing trend over time. EI% of the morning snack, afternoon snack and evening snack all showed an increasing trend over time.

With respect to the average levels of the EI% of eating occasions in different subgroups, generally in each wave, breakfast EI% was higher in female, ≥60 years old and rural participants than those in male, 18–59 years old and urban participants. Lunch EI% was higher in male, 18–59 years old and urban participants than those in female, ≥60 years old and rural participants. Dinner EI% was higher in male, 18–59 years old and urban participants than those in female, ≥60 years old and rural participants. EI%s of all snack occasions were higher in female, 18–59 years old and urban participants than those in male, ≥60 years old and rural participants.

## 4. Discussion

The present study demonstrated that the EI% of eating occasions varied across the day, with dinner EI% being the largest in each wave. Besides, the time-of-day of energy intake has undergone a process of change over the past 27 years, from a skewed time-of-day of energy intake with lunch and dinner EI% increasing and breakfast EI% decreasing in the early waves towards an approximately balanced time-of-day of energy intake in the later waves in this Chinese cohort.

Results showed that, the ranges of breakfast EI%, lunch EI% and dinner EI% were 25.47–29.50%, 34.33–37.02%, and 34.87–37.40% respectively, in the study population over the study period. This meal pattern characterized by energy proportion increasing gradually over the day was similar to those of UK [10], USA [23], Germany [24], Denmark and Netherlands [25], but different from those of France, Switzerland, Italy and Northern Ireland, where the energy proportion of lunch was the largest during the day [26]. The difference in meal pattern could be attributed to cultural and social factors [27,28] and variations in satiety through the day [2]. However, a growing body of evidence has suggested meal pattern’s relationship with health outcomes, with a large energy proportion towards later in the day linked to higher risk of obesity, type 2 diabetes and dyslipidemia [4,29,30,31,32,33]. Therefore, the time-of-day of energy intake demonstrated by the present study could provide critical context to research efforts to investigate the relationship with diet-related health outcomes and to generate specific public health recommendations on meal patterns for the study population.

The results of the secular trend analysis showed that both lunch EI% and dinner EI% went through a slight increase between 1991 and 1997, and the EI% from all snack occasions showed little increase before 2000. Similar to our result, Almoosawi et al. [10] observed an increase of evening EI% from 1982 to 1999 using the 1946 British birth cohort. However, the increase was much higher than that of ours, with an approximately 3.0% increase of evening EI% from 1982 to 1999 in Almoosawi’s study, compared to an approximately 0.4% increase of evening period EI% (dinner + evening snack) from 1991 to 2000 in our study. The large difference between the two studies might be attributed to the different social environments in the two countries. One early study [34] reported that simple lunch and mid-afternoon snacks were often eaten among working British adults due to time scarcity in the daytime, while a big dinner was eaten as an important social event with family members in Britain. By comparison, both lunch and dinner are viewed as main meals in China. The decline of lunch EI% observed in Almoosawi’s study might contribute to the higher increase of evening EI%. A second explanation might be the relatively slow pace of development of the modern food system in China before 2000. Before 2000, only a few supermarkets, convenience stores and fast-food restaurants were opened in big cities in China. The very low proportion of take-away food and snacks had little impact on people’s traditional meal patterns [15].

As shown in the results, with the fast development of a modern food system in China after 2000, all of the EI% from snack occasions showed a rapid increase since 2000. At the same time, a modest 2.5% decrease in both lunch EI% and dinner EI% were observed between 2000 and 2018. Similar to our results, using the National Health and Nutrition Examination Survey (NHANES), Kant et al. [9] found that from NHANES 1997–1975 to NHANES 1999–2002, energy from evening food declined from 45.9% to 44.2%, while energy from snacks increased from 19.7% to 20.8%. Therefore, it can be assumed that the traditional meal pattern of three main meals was replaced by a spread meal pattern with more than three meals during the day. So a certain portion of lunch EI% and dinner EI% was transferred to the afternoon snack and evening snack in the new meal pattern, which could explain the decline of lunch EI% and dinner EI% after 2000 in our study.

As other studies have reported higher proportions of breakfast skipping, snacking, and night-time eating in both developed and developing countries [18,35], we had expected to observe an increase of evening period EI% (dinner + evening snack) from 2000 to 2018, given that the fast development of a modern food system and the westernization of dietary patterns [36] began to take place in China since 2000. However, contrary to our anticipation, the EI% of the evening period (dinner + evening snack) showed a decrease of 1.74% from 2000 to 2018, resulting in a shift of energy distribution based on morning, afternoon and evening periods of approximately 2.5:3.7:3.8 in 2000 to 3.0:3.5:3.5 in 2018. It seemed that a more balanced energy distribution was adopted by our study population as time went by. However, it is of note that the population aging might have an effect on the eating behavior since participants in our study were from a cohort, rather than multiple panel surveys. The percentage of participants aged ≥60 years reached 44.25% in 2018 in our study. Older people are concerned with health, and are less sensitive to hunger or satiety cues than younger people [23], therefore they tend to follow a meal pattern that is more balanced in energy distribution. Another possible explanation might be the increasing percentage of females in this cohort. Females are highly motivated by weight control and tend to diet [37], especially to skip lunch and dinner [38,39,40]. Although both age and gender were controlled for in calculating marginal mean of EI% in the whole participants, the declining trends may be affected by these demographic changes. More trend analyses using panel data might be needed.

Results showed demographic disparities in the rate of change of EI% of eating occasions with time. To the best of our knowledge, no other study has examined demographic disparity in the time trend, so comparison with other studies is not available. As for the average level of EI% of each eating occasion during the study period, generally, females, those ≥60 years old, and the urban population had higher breakfast EI% and lower lunch EI% and dinner EI%, while females, 18–59 years old, and the urban population had higher snack EI%, which were similar to other studies [10,18,23]. All of these results regarding demographic disparities in secular trends or average levels suggested different associations of the time-of-day of energy intake with health outcomes in subpopulations. Therefore, targeted education strategies regarding gender, age, and region in the public health recommendations need to be considered.

This study complemented previous research in that a secular trend of time-of-day of energy intake in Chinese adults over 27 years was described, and potential demographic differences in trends were explored. The long study period enabled us to capture different change profiles in different time frames. Several limitations should be mentioned. First, eating occasions were pre-defined in the 3–24 h recall and were self-identified by participants. Therefore, no objective measure of eating occasion was applied, such as clock time, which might limit the comparability with other studies. Second, participants might under-report food or beverages that were eaten or drunk mindlessly or in a small amount, especially at snacking occasions. The under-reporting of a wide variety of foods was found during dinner by one previous study [41]. Therefore, it is possible that selective under-reporting on specific eating occasions might exist in our study. Since no sufficient data on dietary under-reporting at meals are available, post hoc adjustments for energy intake at meals could not be conducted. More studies are needed to fill this gap.

## 5. Conclusions

In conclusion, the present study demonstrated that time-of-day of energy intake varied throughout the day and that time-of-day of energy intake firstly shifted towards later in the day and then towards a balanced meal pattern over a period of 27 years in a Chinese cohort population. Further studies should attempt to interpret the health implications of such changes of meal pattern and the demographic disparities.

## Figures and Tables

**Table 1:** Cross-sectional characteristics of the study sample in CHNS 1991 to 2018.

Variables	1991	1993	1997	2000	2004	2006	2009	2011	2015	2018
N	6915	7066	7125	7379	8022	8069	8576	10,411	11,846	10,249
Age groups (%)										
18–59 years	86.12	83.75	81.39	79.20	76.58	74.00	70.98	68.67	63.64	55.75
≥60 years	13.88	16.25	18.61	20.80	23.42	26.00	29.02	31.33	36.36	44.25
Gender (%)										
Male	48.04	48.00	49.49	48.80	47.87	47.14	47.95	47.24	46.03	45.18
Female	51.96	52.00	50.51	51.20	52.13	52.86	52.05	52.76	53.97	54.82
Geographical region (%)										
Urban	31.90	32.17	33.45	32.69	34.28	34.06	33.12	38.28	38.08	37.00
Rural	68.10	67.83	66.55	67.31	65.72	65.94	66.88	61.72	61.92	63.00
Educational level (%)										
Low	56.70	54.53	53.39	49.70	44.38	43.02	42.34	36.82	33.05	30.88
Medium	15.33	16.47	17.74	20.38	23.67	26.20	23.86	29.96	34.00	35.90
High	27.97	29.00	28.87	29.92	31.95	30.78	33.80	33.22	32.95	33.22
Marriage status (%)										
Married	82.30	80.88	81.47	79.25	83.96	85.30	85.00	85.13	88.93	87.58
Other status	17.70	19.12	18.53	20.75	16.04	14.70	15.00	14.87	11.07	12.42
Smoking (n, %)										
No	65.03	67.20	67.76	68.71	70.74	72.52	71.39	73.20	76.61	79.27
Yes	34.97	32.80	32.24	31.29	29.26	27.48	28.61	26.80	23.39	20.73
Alcohol drinking (%)										
No	61.45	63.64	63.27	64.66	66.85	67.89	66.65	66.13	72.26	74.75
Yes	38.55	36.36	36.73	35.34	33.15	32.11	33.35	33.87	27.74	25.25
Chronic diseases (%)										
No	96.17	95.33	94.29	91.29	89.06	87.77	83.97	79.69	78.83	75.17
Yes	3.83	4.67	5.71	8.71	10.94	12.23	16.03	20.31	21.17	24.83
Physical activity (Mets/week h, Median [IQR]))	391.75	318.70	310.50	247.50	140.99	139.75	141.5	139.53	95.03	108.85
	(192.00,622.85)	(174.09, 511.25)	(128.60, 506.50)	(409.70, 105.53)	(54.78, 306.50)	(51.80, 300.34)	(56.7, 290.81)	(61.6, 265.13)	(37.00, 198.17)	(47.83, 207.73)
Per capita household income (Yuan, Median [IQR])	3470.64	4424.03	8641.98	9928.35	11,841.87	13,895.51	23,352.17	33,461.90	48,658.90	58,536.59
	(2000.0,5505.6)	(2486.6, 7570.1)	(4767.5, 14,057.6)	(4934.6, 16,838.2)	(5884.5, 21,599.3)	(6783.6, 25,516.0)	(12,186.9, 41,217.6)	(17,196.3, 57,901.2	(21,679.7, 82,758.6)	(25,220.5, 103,982.3)
Urbanicity score (mean [SD])	46.39	48.38	52.69	58.21	62.88	64.83	67.42	70.80	72.51	71.43
	(16.18)	(16.41)	(17.99)	(18.24)	(20.24)	(20.35)	(19.44)	(19.01)	(17.42)	(16.92)
Total energy intake (kcal, mean [SD])	2692. 05	2597.53	2462.54	2421.61	2378.21	2335.37	2321.11	2091.51	2009.22	1988.06
	(695.24)	(698.57)	(707.00)	(735.89)	(772.79)	(765.18)	(734.32)	(716.06)	(717.43)	(692.57)
BMI (kg/m^2^, mean [SD])	21.67	21.91	22.35	22.84	23.11	23.25	23.39	23.95	24.22	24.48
	(2.84)	(2.87)	(3.11)	(3.24)	(3.35)	(3.33)	(3.47)	(4.09)	(3.67)	(3.65)

**Table 2:** Secular trends and marginal mean of energy proportions of eating occasions in study sample from 1991 to 2018.

	1991 (N = 6915)	1993 (N = 7066)	1997 (N = 7125)	2000 (N = 7379)	2004 (N = 8022)	2006 (N = 8069)	2009 (N = 8576)	2011 (N = 10,411)	2015 (N = 11,846)	2018 (N = 10,249)	Time	Time ^2^
Breakfast EI% (marginal mean [SE]) ^1^	26.84 (0.11)	26.32 (0.09)	25.64 (0.07)	25.43 (0.07)	25.55 (0.07)	25.78 (0.06)	26.34 (0.06)	26.86 (0.06)	28.23 (0.07)	29.56 (0.10)	−0.28 ***	0.014 ***
Lunch EI% (marginal mean [SE]) ^1^	36.86 (0.11)	36.97 (0.09)	37.05 (0.07)	36.98 (0.07)	36.72 (0.07)	36.52 (0.06)	36.12 (0.06)	35.80 (0.06)	35.02 (0.07)	34.31 (0.09)	0.07 ***	−0.006 ***
Dinner EI% (marginal mean [SE]) ^1^	37.19 (0.10)	37.31 (0.08)	37.40 (0.06)	37.35 (0.06)	37.13 (0.06)	36.94 (0.06)	36.58 (0.06)	36.28 (0.05)	35.54 (0.06)	34.86 (0.08)	0.07 ***	−0.006 ***
Moring snack EI% (marginal mean [SE]) ^2^	0.01 (0.001)	0.01 (0.001)	0.03 (0.001)	0.04 (0.002)	0.07 (0.002)	0.10 (0.003)	0.14 (0.003)	0.18 (0.004)	0.27 (0.005)	0.37 (0.008)	0.16 ***	−0.00004
Afternoon snack EI% (marginal mean [SE]) ^2^	0.02 (0.002)	0.03 (0.002)	0.06 (0.002)	0.09 (0.002)	0.15 (0.003)	0.18 (0.003)	0.24 (0.004)	0.28 (0.005)	0.36 (0.006)	0.43 (0.008)	0.19 ***	−0.002 ***
Evening snack EI% (marginal mean [SE]) ^2^	0.03 (0.002)	0.05 (0.002)	0.08 (0.002)	0.11 (0.002)	0.17 (0.003)	0.20 (0.004)	0.25 (0.004)	0.29 (0.005)	0.36 (0.006)	0.42 (0.008)	0.14 ***	−0.002 ***
Moring period EI% (marginal mean [SE]) ^1^	26.98 (0.11)	26.47 (0.09)	25.81 (0.07)	25.64 (0.07)	25.83 (0.07)	26.12 (0.07)	26.77 (0.06)	27.36 (0.06)	28.90 (0.07)	30.37 (0.10)	−0.29 ***	0.015 ***
Afternoon period EI% (marginal mean [SE]) ^1^	37.07 (0.11)	37.23 (0.09)	37.39 (0.07)	37.40 (0.07)	37.26 (0.07)	37.13 (0.06)	36.84 (0.06)	36.59 (0.06)	35.96 (0.07)	35.37 (0.09)	0.09 ***	−0.006 ***
Evening period EI% (marginal mean [SE]) ^1^	37.46 (0.10)	37.64 (0.08)	37.85 (0.06)	37.90 (0.06)	37.80 (0.06)	37.69 (0.06)	37.43 (0.06)	37.21 (0.05)	36.62 (0.06)	36.06 (0.08)	0.10 ***	−0.006 ***

^1^ Multilevel linear mixed model was applied. ^2^ Log-transformation was done to improve normality before multilevel Tobit regression model was applied. Models were adjusted for age, gender, educational level, geographical region, total physical activity, smoking, alcohol drinking, annual per capita household income, community urbanicity index, chronic disease history, total energy intake, and BMI. *** *p* < 0.001.

**Table 3:** Interaction between time and demographic variables.

Interaction Terms	Breakfast EI% ^1^	Lunch EI% ^1^	Dinner EI% ^1^	Morning Snack EI% ^2^	Afternoon Snack EI% ^2^	Evening Snack EI% ^2^
Gender (male = 0, female = 1)						
Gender × time	0.07 *	−0.03	−0.03	0.05 *	0.02	0.006
Gender × time ^2^	−0.002	0.0005	0.00004	−0.0006	−0.0001	−0.000001
Age group (18–59 years = 0, ≥60 years = 1)						
Age group × time	0.09 *	−0.02	−0.09 **	0.03	0.12 ***	0.07 ***
Age group × time ^2^	−0.002	0.0008	0.001	0.0003	−0.004 ***	−0.002 ***
Geographic region (urban = 0, rural = 1)						
Geographic region × time	−0.24 ***	0.23 ***	0.02	−0.07 **	0.04	0.01
Geographic region × time ^2^	0.005 ***	−0.006 ***	0.003 **	0.002 *	−0.001 *	0.0007

^1^ Multilevel linear mixed model was applied. ^2^ Log-transformation was done to improve normality before multilevel Tobit regression model was applied. Models were adjusted for age, gender, educational level, geographical region, total physical activity, smoking, alcohol drinking, annual per capita household income, community urbanicity index, chronic disease history, total energy intake, and BMI. *** *p* < 0.001, ** *p* < 0.01, * *p* < 0.05.

**Table 4:** Trend analysis of energy proportions of eating occasions by gender, age-group and location in study sample from 1991 to 2018.

	Breakfast EI% ^1^		Lunch EI% ^1^		Dinner EI% ^1^		Morning Snack EI% ^2^		Afternoon Snack EI% ^2^		Evening Snack EI% ^2^	
	Time	Time ^2^	Time	Time ^2^	Time	Time ^2^	Time	Time ^2^	Time	Time ^2^	Time	Time ^2^
Gender												
Male	−0.31 ***	0.015 ***	0.08 ***	−0.006 ***	0.08 ***	−0.005 ***	0.14 ***	−0.00002	0.19 ***	−0.003 ***	0.14 ***	−0.002 ***
Female	−0.26 ***	0.014 ***	0.05 **	−0.006 ***	0.06 **	−0.006 ***	0.17 ***	−0.0001	0.20 ***	−0.002 ***	0.15 ***	−0.001 ***
Age group												
18–59 years	−0.27 ***	0.014 ***	0.05 **	−0.006 ***	0.07 ***	−0.006 ***	0.17 ***	0.0001	0.18 ***	−0.001 ***	0.13 ***	−0.001 ***
≥60 years	−0.26 ***	0.014 ***	0.07	−0.006 ***	0.04	−0.005 ***	0.13 ***	−0.00005	0.25 ***	−0.004 ***	0.19 ***	−0.003 ***
Geographic region												
Urban	−0.21 ***	0.012 ***	−0.06 *	−0.003 **	0.18 ***	−0.010 ***	0.17 ***	−0.0006	0.15 ***	−0.001 ***	0.11 ***	−0.001 ***
Rural	−0.34 ***	0.016 ***	0.14 ***	−0.008 ***	0.04 **	−0.004 ***	0.14 ***	0.0007	0.23 ***	−0.003 ***	0.18 ***	−0.002 ***

^1^ Multilevel linear mixed model was applied. ^2^ Log-transformation was done to improve normality before multilevel Tobit regression model was applied. Models were adjusted for age, gender, educational level, geographical region, total physical activity, smoking, alcohol drinking, annual per capita household income, community urbanicity index, chronic disease history, total energy intake, and BMI. *** *p* < 0.001, ** *p* < 0.01, * *p* < 0.05.

## Data Availability

The datasets generated during and/or analyzed during the current study are available from the corresponding authors (B.Z.) on reasonable request.

## Supplementary Materials

The following supporting information can be downloaded at: https://www.mdpi.com/article/10.3390/nu14102019/s1, Table S1: Marginal means of energy proportion of breakfast, lunch, dinner by gender age-group and location; Table S2: Marginal means of energy proportion of morning snack, afternoon snack, evening snack by gender age-group and location.
