# Supplementary material for: Secular Trends in Time-of-Day of Energy Intake in a Chinese Cohort
Source: Nutrients. 2022 May 11;14(10):2019. doi: 10.3390/nu14102019 (PMC9146504; doi:10.3390/nu14102019)
Supplement: Supplementary file 1 [file nutrients-14-02019-s001.zip › nutrients-1664246-supplementary.pdf]

Table S1. Marginal means of energy proportion of breakfast, lunch, dinner by gender, age-group and location

	1991 Year (N = 6915)	1993 Year (N = 7066)	1997 Year (N = 7125)	2000 Year (N = 7379)	2004 Year (N = 8022)	2006 Year (N = 8069)	2009 Year (N = 8576)	2011 Year (N = 10,411)	2015 Year (N = 11,846)	2018 Year (N = 10,249)
Breakfast EI% (marginal mean [SE])[1]										
Male	26.8(0.16)	26.2(0.13)	25.4(0.10)	25.1(0.10)	25.2(0.10)	25.4(0.09)	25.9(0.09)	26.4(0.09)	27.8(0.11)	29.1(0.14)
Female	26.9(0.16)	26.4(0.13)	25.8(0.10)	25.7(0.09)	25.9(0.09)	26.1(0.09)	26.7(0.09)	27.2(0.08)	28.6(0.10)	30.0(0.13)
18–59 years	26.6(0.12)	26.1(0.10)	25.5(0.08)	25.3(0.07)	25.5(0.07)	25.7(0.07)	26.3(0.07)	26.8(0.07)	28.2(0.09)	29.6(0.12)
≥60 years	26.9(0.28)	26.4(0.23)	25.8(0.16)	25.6(0.14)	25.8(0.13)	26.0(0.13)	26.6(0.12)	27.1(0.11)	28.5(0.11)	29.8(0.15)
Urban	24.7(0.19)	24.4(0.15)	23.9(0.11)	23.8(0.10)	24.1(0.10)	24.3(0.10)	24.9(0.09)	25.4(0.09)	26.8(0.10)	28.0(0.14)
Rural	28.0(0.14)	27.4(0.12)	26.5(0.09)	26.2(0.09)	26.3(0.09)	26.5(0.08)	27.0(0.08)	27.5(0.08)	29.0(0.10)	30.4(0.12)
Lunch EI% (marginal mean [SE])[1]										
Male	36.9(0.16)	37.0(0.13)	37.2(0.10)	37.1(0.10)	36.9(0.10)	36.7(0.09)	36.4(0.09)	36.0(0.09)	35.3(0.11)	34.6(0.14)
Female	36.8(0.16)	36.9(0.13)	36.9(0.10)	36.8(0.09)	36.5(0.09)	36.3(0.09)	35.9(0.09)	35.6(0.09)	34.8(0.10)	34.1(0.13)
18–59 years	37.2(0.12)	37.3(0.10)	37.3(0.08)	37.2(0.07)	36.8(0.07)	36.6(0.07)	36.2(0.07)	35.8(0.07)	35.0(0.09)	34.3(0.12)
≥60 years	36.3(0.29)	36.4(0.24)	36.5(0.17)	36.4(0.14)	36.2(0.13)	36.1(0.13)	35.7(0.12)	35.4(0.11)	34.7(0.11)	34.1(0.15)
Urban	38.2(0.18)	38.0(0.14)	37.7(0.10)	37.4(0.10)	36.9(0.10)	36.7(0.09)	36.2(0.09)	35.9(0.09)	35.2(0.10)	34.7(0.14)
Rural	36.2(0.14)	36.5(0.12)	36.8(0.10)	36.8(0.09)	36.7(0.09)	36.5(0.09)	36.1(0.08)	35.8(0.08)	34.9(0.10)	34.0(0.13)
Dinner EI% (marginal mean [SE])[1]										
Male	37.4(0.14)	37.6(0.12)	37.7(0.09)	37.7(0.09)	37.5(0.08)	37.4(0.08)	37.1(0.08)	36.8(0.08)	36.2(0.09)	35.6(0.13)
Female	37.0(0.14)	37.1(0.12)	37.1(0.09)	37.0(0.08)	36.8(0.08)	36.5(0.08)	36.1(0.08)	35.8(0.08)	35.0(0.09)	34.2(0.12)
18–59 years	37.4(0.11)	37.5(0.09)	37.6(0.07)	37.6(0.07)	37.3(0.07)	37.1(0.07)	36.8(0.06)	36.5(0.06)	35.7(0.08)	35.0(0.11)
≥60 years	37.2(0.25)	37.2(0.20)	37.2(0.14)	37.1(0.13)	36.8(0.12)	36.6(0.11)	36.2(0.10)	35.8(0.10)	35.1(0.10)	34.4(0.13)
Urban	37.1(0.18)	37.4(0.14)	37.8(0.10)	37.9(0.09)	37.7(0.09)	37.5(0.09)	37.1(0.09)	36.6(0.08)	35.6(0.10)	34.5(0.13)
Rural	37.1(0.12)	37.2(0.10)	37.2(0.08)	37.1(0.08)	36.9(0.07)	36.7(0.07)	36.4(0.07)	36.2(0.07)	35.6(0.08)	35.0(0.11)

[1] Multilevel linear mixed model was applied. Models were adjusted for age, gender, educational level, geographical region, total physical activity, smoking, alcohol drinking, annual per capita household income, community urbanicity index, chronic disease history, total energy intake, and BMI.

Table S2. Marginal means of energy proportion of morning snack, afternoon snack, evening snack by gender, age-group and location

	1991 Year (N = 6915)	1993 Year (N = 7066)	1997 Year (N = 7125)	2000 Year (N = 7379)	2004 Year (N = 8022)	2006 Year (N = 8069)	2009 Year (N = 8576)	2011 Year (N = 10,411)	2015 Year (N = 11,846)	2018 Year (N = 10,249)
Morning snack EI% (marginal mean [SE])[1]										
Male	0.01(0.002)	0.02(0.002)	0.03(0.002)	0.04(0.002)	0.07(0.003)	0.09(0.004)	0.12(0.005)	0.15(0.005)	0.22(0.007)	0.30(0.011)
Female	0.01(0.001)	0.01(0.002)	0.03(0.002)	0.04(0.002)	0.08(0.003)	0.11(0.004)	0.16(0.005)	0.20(0.006)	0.32(0.008)	0.44(0.012)
18–59 years	0.01(0.001)	0.01(0.001)	0.03(0.002)	0.04(0.002)	0.07(0.003)	0.09(0.003)	0.14(0.004)	0.18(0.005)	0.29(0.007)	0.40(0.012)
≥60 years	0.01(0.004)	0.02(0.004)	0.03(0.004)	0.05(0.004)	0.08(0.004)	0.10(0.005)	0.14(0.006)	0.18(0.007)	0.26(0.008)	0.35(0.012)
Urban	0.01(0.002)	0.02(0.003)	0.04(0.003)	0.06(0.003)	0.10(0.004)	0.13(0.005)	0.19(0.006)	0.24(0.007)	0.35(0.009)	0.46(0.014)
Rural	0.01(0.001)	0.01(0.001)	0.02(0.002)	0.04(0.002)	0.06(0.002)	0.08(0.003)	0.12(0.004)	0.15(0.005)	0.24(0.007)	0.34(0.011)
Afternoon snack EI% (marginal mean [SE]) [1]										
Male	0.02(0.002)	0.03(0.003)	0.05(0.003)	0.08(0.003)	0.13(0.004)	0.16(0.005)	0.21(0.006)	0.24(0.006)	0.30(0.008)	0.35(0.011)
Female	0.02(0.002)	0.03(0.003)	0.06(0.003)	0.10(0.003)	0.16(0.004)	0.20(0.005)	0.27(0.006)	0.32(0.007)	0.42(0.008)	0.50(0.012)
18–59 years	0.02(0.002)	0.03(0.002)	0.05(0.002)	0.08(0.003)	0.14(0.003)	0.17(0.004)	0.23(0.005)	0.28(0.006)	0.38(0.008)	0.47(0.011)
≥60 years	0.01(0.003)	0.02(0.004)	0.05(0.006)	0.09(0.006)	0.17(0.006)	0.21(0.007)	0.26(0.008)	0.30(0.009)	0.35(0.009)	0.37(0.012)
Urban	0.04(0.005)	0.05(0.005)	0.09(0.005)	0.13(0.005)	0.20(0.006)	0.24(0.007)	0.31(0.008)	0.36(0.008)	0.47(0.010)	0.56(0.015)
Rural	0.01(0.002)	0.02(0.002)	0.04(0.002)	0.07(0.003)	0.12(0.003)	0.16(0.004)	0.21(0.005)	0.25(0.006)	0.32(0.007)	0.37(0.010)
Evening snack EI% (marginal mean [SE]) [1]										
Male	0.03(0.003)	0.04(0.003)	0.07(0.003)	0.11(0.003)	0.16(0.004)	0.18(0.005)	0.23(0.006)	0.26(0.006)	0.33(0.008)	0.38(0.011)
Female	0.04(0.003)	0.05(0.003)	0.08(0.003)	0.12(0.004)	0.18(0.004)	0.21(0.005)	0.26(0.006)	0.30(0.006)	0.39(0.008)	0.45(0.011)
18–59 years	0.04(0.003)	0.05(0.003)	0.08(0.003)	0.11(0.003)	0.17(0.004)	0.20(0.004)	0.25(0.005)	0.29(0.006)	0.38(0.007)	0.46(0.011)
≥60 years	0.02(0.004)	0.03(0.005)	0.07(0.006)	0.10(0.006)	0.16(0.006)	0.19(0.007)	0.24(0.008)	0.27(0.008)	0.32(0.009)	0.34(0.012)
Urban	0.10(0.008)	0.12(0.007)	0.18(0.006)	0.23(0.006)	0.31(0.007)	0.34(0.008)	0.40(0.008)	0.43(0.009)	0.50(0.010)	0.54(0.013)
Rural	0.02(0.002)	0.02(0.002)	0.04(0.002)	0.07(0.002)	0.11(0.003)	0.14(0.004)	0.19(0.005)	0.23(0.005)	0.32(0.007)	0.38(0.011)

[1] Log-transformation was done to improve normality before multilevel Tobit regression model was applied. Models were adjusted for age, gender, educational level, geographical region, total physical activity, smoking, alcohol drinking, annual per capita household income, community urbanicity index, chronic disease history, total energy intake, and BMI.
